# Chemical Composition and Acaricidal Activity of the Essential Oils of Some Plant Species of* Lamiaceae* and* Myrtaceae* against the Vector of Tropical Bovine Theileriosis:* Hyalomma scupense* (syn.* Hyalomma detritum*)

**DOI:** 10.1155/2019/7805467

**Published:** 2019-02-07

**Authors:** Somia Djebir, Samir Ksouri, Mohamed Trigui, Slim Tounsi, Awatif Boumaaza, Youssef Hadef, Ahmed Benakhla

**Affiliations:** ^1^Institute of Veterinary Science, University Chadli Bendjedid El-Tarf, BP 73, El-Tarf 36000, Algeria; ^2^Department of Biology, Faculty of Nature and Life Sciences and Earth Sciences and the Universe, University of 8 Mai 1945 Guelma, BP 401 Guelma 24000, Algeria; ^3^Research Unit “Coastal and Urban Environments” University of Sfax, Sfax Preparatory Engineering Institute, BP 1172-3018 Sfax, Tunisia; ^4^Laboratoire des Biopesticides, Centre de Biotechnologie de Sfax (CBS), P.B. 1177, 3018 Sfax, Université de Sfax, Tunisia; ^5^Laboratory of Analytical Chemistry, Department of Pharmacy, Faculty of Medicine, University Badji Mokhtar of Annaba, Route Zaafrania B.P. 205 Annaba 23000, Algeria

## Abstract

The present study aimed to investigate the acaricidal properties of six essential oils. They were extracted from some plant species (*Lamiaceae* and* Myrtaceae*) using the technique of hydrodistillation with the Clevenger apparatus. The chemical compositions of the essential oils under study were determined by gas chromatography–mass spectrometer (GC-MS). An Adult Immersion Test (AIT) and a Larval Immersion Test (LIT) were used to evaluate the acaricidal activity of these essential oils against the adults and larvae of* Hyalomma scupense*. GC-MS analysis showed the major constituents of each essential oil: 25.49% of *α*-thujone (lavender); 46.82% of carvacrol (oregano); 78.78% of carvacrol (thyme); 40.27% of 1,8-cineole (blue gum); 17.45% of p-cymene (river red gum); and 26.96% of 1,8-cineole (rosemary). The biotests on the essential oils revealed that they inhibit the reproduction of* H. scupense* engorged females at a rate of 100 % with doses of 0.781 *μ*l/ml of rosemary, 1.562 *μ*l/ml of thyme, 3.125 *μ*l/ml of lavender and oregano, and 6.250 *μ*l/ml of blue gum and river red gum. After a treatment that lasted for 24 hours, essential oils showed a larvicidal activity with respective values of lethal concentrations (LC): LC_50_, LC_90_, and LC_95_ (0.058, 0.358, and 0.600 *μ*l/ml for thyme; 0.108, 0.495, and 0.761 *μ*l/ml for rosemary; 0.131, 0.982, and 1.740 *μ*l/ml for oregano; 0.155, 2.387, and 5.183 *μ*l/ml for blue gum; 0.207, 1.653, and 2.978 *μ*l/ml for river red gum; and 0.253, 2.212, and 4.092 *μ*l/ml for lavender). This is the first report on the acaricidal activity of these essential oils against* H. scupense.* The results obtained showed that the essential oils with chemotype carvacrol, 1,8-cineole, *α*-thujone, and p-cymene are highly acaricidal, and they can be used for ticks control. However, further studies on their toxicity in nontarget organisms are required.

## 1. Introduction

Ticks and the diseases they transmit have long been recognized as one of the major constraints of livestock development in various countries. Ticks feeding on domestic animals can result in various adverse effects including anemia, paralysis, toxicosis, decreased quality of the leather, and transmission of many diseases of diverse etiology. The causative agent of these diseases can be a virus, rickettsia, bacterium, or protozoan [1]. Therefore, the damage directly caused by ticks (and induced pathology) is a serious animal health problem that can significantly reduce overall livestock productivity.

In Algeria, cattle herds cost a heavy price if infected with piroplasmosis, a major tick transmitted disease of livestock in the country [2]. Another tick-borne disease, tropical bovine theileriosis transmitted by* Hyalomma scupense* (syn.* Hyalomma detritum*), is also present in this area. This species has been the most frequently recorded from several surveys conducted in Algeria [3, 4]. Many approaches have been used for tick management such as biological control using pathogens or predators, pheromone-assisted control, herbal pour-on or dip preparations including green manufactured nanoparticles [5], and vaccination [6]. Acaricides and repellents are still regarded as the easiest method for control but applications involve several drawbacks like cost, toxicity, waiting times, and acaricide resistance [7]. In Algeria, the most widely used means to control cattle ticks are conventional acaricides. Despite of the absence of studies on the chemoresistance of marketed acaricides, Algerian veterinary practitioners have observed resistance to these compounds in recent years. Therefore, it is prudent that effective compounds be discovered and evaluated for their acaricidal potential. In this study, we investigated the effectiveness of various essential oils (EO) against* H. scupense* obtained from six aromatic plant species:* Eucalyptus camaldulensis* Dehnh (river red gum),* Eucalyptus globulus* Labill (blue gum),* Lavandula stoechas* L. (lavender),* Origanum floribundum* Munby (oregano),* Rosmarinus officinalis* L. (rosemary), and* Thymus capitatus* L. (thyme).

## 2. Materials and Methods

### 2.1. Plant Materials

During April through July 2015, leaves and flowering tops were collected from their natural habitats from Wilaya of Guelma: lavender, Ain Safra region of Djebel Maouna, latitude: 36.403237, longitude: 7.387801; oregano, Djebel Haouara region, latitude: 36.544436, longitude: 7.523108; thyme, Ouled Chiha region of Hammam Ouled Ali, latitude: 36.588422, longitude: 7.467377; and blue gum and river red gum, Djebel Beni Salah area, latitude: 36.476148, longitude: 7.854270. Leaves and flowering tops of rosemary were harvested on April 2015 from the Wilaya of Tebessa in Ouenza region (Gora Range) latitude: 35.917372, longitude: 8.127908.

### 2.2. Essential Oils Extraction and Analyses

Extraction of EO from plant parts was carried out by hydrodistillation for three hours using a Clevenger-type hydrodistillation apparatus. GC-MS analysis of the oils was performed on an HP-MS HP Model 6980 inert MSD (Agilent Technologies, USA), with HP-5MS column (30 × 0.25 mm ID × 0.25 *μ*m film thickness). Temperature of the injector was maintained at 280°C. Oven temperature was maintained at 60°C for 1 min and then increased to 280°C at 5°C/min and remained constant at this temperature for 8 min. Flow rate of the helium carrier was 1 ml/min and split mode 1/100 was used. Identification of components in the EO was accomplished by comparison of their Kovats index and GC mass fragmentation with those of Wiley Mass Spectral data (Agilent Technologies 7th edition, Inc.) and NIST 05 MS library data. Each analysis was executed in duplicate.

### 2.3. Ticks Collection

Engorged females of* H. scupense*, with an average weight of 0.50 g, were collected from naturally infested cattle. Ticks were washed with 2% sodium hypochlorite solution and were then rinsed with distilled water before being dried with paper towels [8]. Engorged females used for Adult Immersion Test (AIT) and larvae for Larval Immersion Test (LIT) were tested on the same day of collection.

### 2.4. Bioassays

#### 2.4.1. Adult Immersion Test (AIT)

Essential oils concentrations used in Adult Immersion Tests (AIT) were prepared in series diluted in Tween 80 at 2% ranging from 12.5 to 0.097*μ*l/ml. Engorged females were each immersed in a concentration for 5 minutes and were then removed and dried with paper towels. Ticks were individually incubated at 27°C and 90% relative humidity with a 12:12 light: dark photoperiod until the end of oviposition [9]. Eggs of each female were weighed and transferred to tubes that were covered with Tissue-Non-Tissue (TNT) fabric (Inotis: LICIAL®, spunlace nonwovens, Algeria) and fastened by elastic bands. Each bioassay was repeated three times. A similar group of ticks as untreated controls was prepared in parallel by the immersion of ticks in 2% Tween 80. The oviposition rates were assessed after the end of oviposition. Reproductive efficiency and reproduction inhibition were calculated using the following equations [10]:

(1) OR=EWIFWOR%=ORcontrol−ORtreatedORcontrol×100RI%=REcontrol−REtreatedREcontrol×100RE=EWIFW×%E

where OR is the oviposition rate, IFW is the initial female weight, EW is the egg weight, E is egg eclosion, RE is the reproductive efficiency, and RI is reproduction inhibition.

#### 2.4.2. Larval Immersion Test (LIT)

Larval immersion bioassays utilized the syringe test technique [9] to evaluate the larvicidal activity of the EO. Approximately 200 eggs (0.01 g) were transferred to a 2.5 ml open-end syringe whose plunger was withdrawn to the line of 2 ml. The syringe was then sealed with a TNT fabric and incubated at 27°C with 90% RH in darkness. Bioassays started 14 days after eclosion.

Essential oils were serially diluted in 2% Tween 80 to obtain ten concentrations ranging from 12.5 *μ*l/ml to 0.024 *μ*l/ml. Larvae were exposed to 2 ml of a concentration per syringe replicated twice. Larvae were exposed to each concentration for 5 min. Mortality in one syringe was recorded at 24 h, while larval mortality in the second syringe was recorded at 6 days. Each bioassay was repeated three times. After removing the TNT parts and emptying the syringes contents, the number of the living and dead larvae was counted in both syringes incubated at 24 h and 6 days. Control groups were handled similarly and were exposed to 2% Tween 80 solution only.

### 2.5. Statistical Analysis

The results of mean oviposition rate, egg eclosion rate, reproduction efficiency, and reproduction inhibition for adult ticks (*H. scupense*) were subjected to nonparametric tests using the Kruskal Wallis test [11]. Estimated LC_50_, _90_, and _95_ of larvae of each EO were determined at 24 h and 6 days. Lethal concentration data were transformed according to Finney's probit analysis method [12]. The Tukey test was used to identify differences between mean values of the LC_50_, LC_90_, and LC_95_ which were obtained at 24 h and after 6 days for each EO. All these statistical analyses were performed using the Social Science Statistics Software (SPSS) for Windows, version 20.0. [13] (IBM Corp. released 2011 IBM SPSS Statistics for Windows, Version 20.0. Armonk, NY: IBM Corp.)

## 3. Results

According to the results of GC-MS chromatographic analysis (Table 1), the major components of river red gum EO are p-cymene, (+) spathulenol, (E, E) –farnesol, *α*-pinene, cuminic aldehyde, 1-phellandrene, sabinene, carvacrol, and p-cymen-7-ol. For blue gum EO, 1,8-cineole, *α*-pinene, viridiflorol, camphene, d-pinocarvone, and (+) - aromadendrene are the major components. The major constituents of lavender EO were *α*-thujone, camphor, camphene, D-fenchyl alcohol, l-bornyl acetate, terminalol L, dl -limonene, *α*-pinene, and linalool L. The main chemical components of oregano EO were carvacrol, p-cymene, and *γ*-terpinene followed by *β*-myrcene, o-cymene, thymol, trans-caryophyllene, *α*-pinene, and *α*-terpinene. Essential oil of rosemary contained 1,8-cineole and l-camphor as major components; other recorded compounds were *α*-pinene, borneol L, camphene, *α*-terpineol, *β*-pinene, trans-caryophyllene, l-bornyl acetate, *β*-myrcene, and *γ*-terpinene. In addition to the major components of thyme EO (carvacrol, p-cymene, and *γ*-terpinene), other compounds from fractionation included transcaryophyllene, m-thymol, *β*-myrcene, and *α*-terpinene.

**Figure 1:**
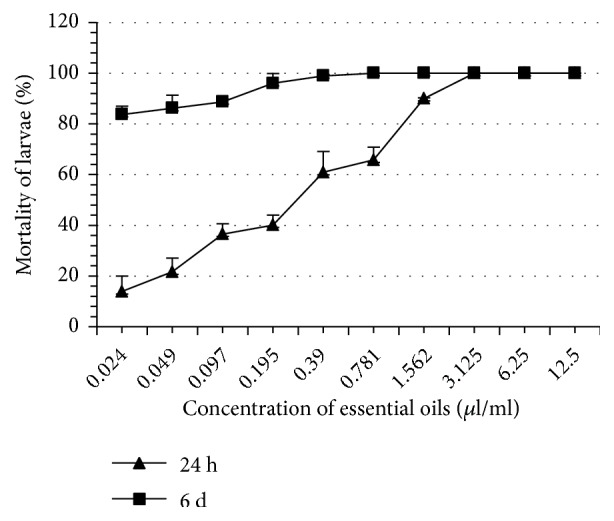
Percentage mortality for* H*.* scupense* larvae exposed to concentrations of river red gum essential oil.

**Figure 2:**
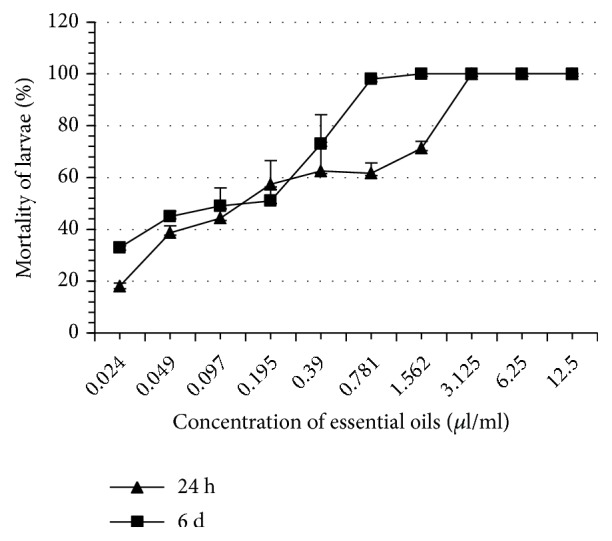
Percentage mortality for* H*.* scupense* larvae exposed to concentrations of blue gum essential oil.

**Figure 3:**
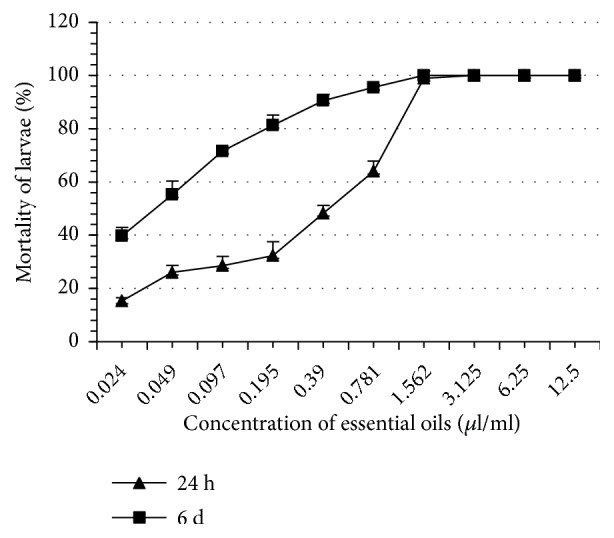
Percentage mortality for* H*.* scupense* larvae exposed to concentrations of lavender essential oil.

**Figure 4:**
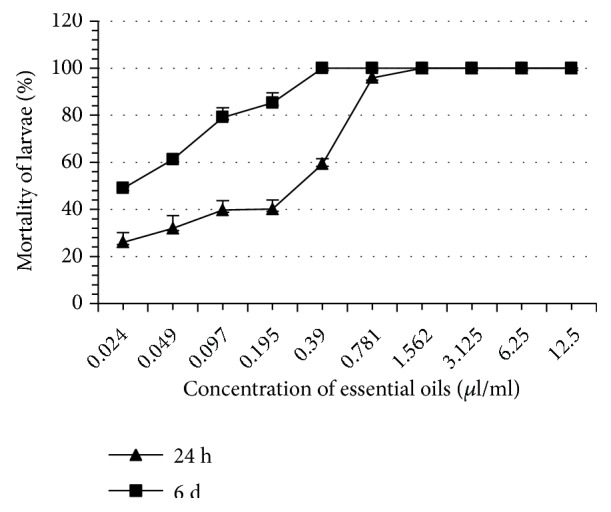
Percentage mortality for* H*.* scupense* larvae exposed to concentrations of oregano essential oil.

**Figure 5:**
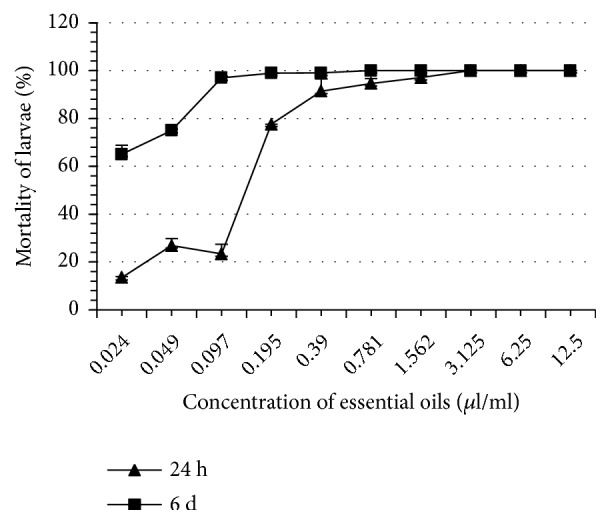
Percentage mortality for* H*.* scupense* larvae exposed to concentrations of rosemary essential oil.

**Figure 6:**
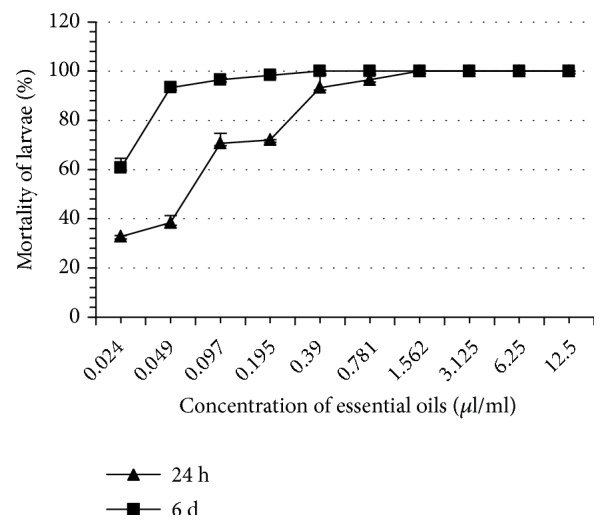
Percentage mortality for* H*.* scupense* larvae exposed to concentrations of thyme essential oil.

Thyme EO completely inhibited tick oviposition at 100% at a concentration of 1.562 *μ*l/ml whereas the other EO induced this same effect at greater concentrations ranging from 3.125 to 6.25 *μ*l/ml (Table 2). At the same concentration (1.562 *μ*l/ml), thyme EO completely inhibited reproduction of* H. scupense*. This concentration is significantly lower than what was recorded with the other EO (3.125 - 6.25 *μ*l/ml). The eclosion rates of the eggs in all treatments were significantly different as compared to controls (p < 0.05). Note that thyme and rosemary EO are more inhibitors of eclosion than other EO such as those of river red gum and blue gum. At 24 hours, thyme EO proved to be the most larvicidal, whereas the least larvicidal was blue gum EO. On the 6th day, the most and the least larvicidal EO remained the same as those obtained in 24 h (Table 3, Figures 1–6). We observed no mortality in control groups in LIT and AIT.

## 4. Discussion

In general, most of the major components of the EO tested in our study (carvacrol, 1,8-cineole, *α*-thujone, borneol L, *α*-pinene, p-cymene, and *α*-terpinene) have been identified as major acaricidal agents around the world [14–20]. Acaricide and insecticidal properties of other Thymus species have been noted by several authors [21, 22]. Many other studies on the acaricidal properties of EO extracted from other Origanum species have been carried out by Ramzi et al. [23], Koc et al. [24], Cetin et al. [15], and Coskun et al. [16]. Moreover, the larvicidal activity of *α*-pinene (from river red gum) has also been documented against* Aedes aegypti*,* Aedes albopictus* [25], and* Anopheles stephensi* [26]. In our study, 1,8-cineole from rosemary and blue gum EO exhibited excellent acaricidal activity against* H. scupense*. Indeed, according to Pirali-Kheirabadi et al. [27], this EO showed an ovicidal, larvicidal, and adulticidal activity against* Rhipicephalus annulatus*. Furthermore, Martinez-Velazquez et al. [28] asserted that the EO of rosemary plant is lethal to another tick species (*Rhipicephalus microplus*). We found significant acaricidal activity of *α*-thujone in lavender EO on the reproductivity of adults as well as larvicidal action against* H. scupense*.

As far as the present study is concerned, we have noticed that each EO we evaluated for acaricidal activity was composed of a combination of 14 to 37 chemical compounds. This may indicate that associations between constituent compounds should be studied mainly in terms of possible additive, synergistic, or even probable antagonistic properties.

## 5. Conclusion

We found excellent acaricidal activity of several EO constituents that were evaluated against* H. scupense* in this study. Our results indicate that these compounds may provide real alternatives to the current conventional acaricidal products, especially against resistant tick populations. However, further investigations are warranted including toxicological studies on nontarget species regarding the usefulness of these components for tick control.

## Figures and Tables

**Table 1:** Chemical composition of the essential oils tested.

No.	Compounds^a^	KI ^b^	Area (%)					
			*EC * ^c^	*EG * ^d^	*LS * ^e^	*OF * ^f^	*RO * ^g^	*TC * ^h^
1	Tricyclene	921	-	-	0.66	-	0.15	-
2	*α*-thujene	928	-	-	-	0.51	-	0.33
3	*α*-pinene	941	4.41	14.23	1.75	1,93	12.06	0.71
4	camphene	953	-	4.60	7.31	0.39	6.39	-
5	sabinene	977	2.43	-	-	-	-	-
5	*β*-pinene	978	-	0.35	0.08	0.22	3.61	-
6	*β*-myrcene	992	0.76	-	-	3.57	2.23	1.24
7	*α*-phellandrene	998	4.04	-	-	-	-	0.22
8	Δ,3-carene	1004	-	-	-	-	-	1.01
9	*α*-terpinene	1015	-	-	0.10	1.37	-	1.15
10	p-cymene	1018	17.45	-	-	18.35	-	6.62
11	o-cymene	1026	-	-	-	3.53	-	-
12	dl-limonene	1030	-	-	2.13	-	-	-
13	*β*-ocimene	1040	0.14	-	-	0.07	-	-
14	d-pinocarvone	1042	-	4.31	-	-	-	-
15	1,8-cineole	1046	-	40.27	-	-	26.90	-
16	(E)-ocimene	1054	0.08	-	-	-	-	-
17	*α*-terpinolene	1063	0.76	0.46	-	-	0.57	-
18	*γ*-terpinene	1065	0.84	0.25	0.10	11.32	1.37	3.96
19	trans-sabinene hydrate	1070	-	-	-	-	0.03	-
20	*α*-terpinolene	1088	-	-	-	0.32	-	-
21	*α*-thujone	1099	-	-	25.49	-	-	-
22	linalool L	1102	0.64	-	1.43	0.50	0.41	-
23	d-fenchyl alcohol	1139	-	-	6.85	-	-	-
24	l-camphor	1152	-	-	20.06	-	19.00	-
25	pinocarvone	1165	-	-	0.18	-	-	-
26	borneol L	1168	-	-	3.99	-	11.76	-
27	4-terpinenol	1178	-	0.39	0.83	-	-	0.54
28	p-cymene-8-ol	1182	-	-	0.94	-	-	-
29	*α*-terpineol	1190	-	-	-	-	5.77	-
30	myrtenal	1193	-	-	0.68	-	-	-
31	myrtenol	1195	-	0.28	0.56	-	-	-
32	verbenone	1205	-	-	0.88	-	-	-
33	fenchyl acetate	1210	-	-	0.97	-	-	-
34	trans-(+)-carveol	1212	0.16	0.51	-	-	-	-
35	*β*-citronellol	1217	-	-	0,36	-	0.09	-
36	isobornyl formate	1233	-	-	0,13	-	-	-
37	pulegone	1237	-	-	-	-	0.26	-
38	cuminic aldehyde	1240	4.29	-	-	-	-	-
39	l-carvone	1241	-	-	0.51	-	-	-
40	carvacrol methyl ether	1244	-	0.30	0.96	-	0.09	-
41	citrol	1255	-	0.11	-	-	-	-
42	piperitone	1258	0.57	-	-	-	-	-
43	l-bornyl acetate	1285	-	0.08	5.51	-	3.00	-
44	thymol	1286	-	-	-	2.04	0.10	2.14
45	p-cymen-7-ol	1291	1.56	-	-	-	-	-
46	carvacrol	1299	1.59	-	-	46.82	0.36	78.78
47	piperitenone	1339	-	-	-	1.18	0.16	-
48	*γ*-pyronene	1345	0.92	-	-	-	-	-
49	*α*-cubebene	1351	-	-	0.09	-	-	-
50	eugenol	1359	-	-	-	-	0.05	-
51	(+)-cyclosativene	1362	-	-	0.25	-	-	-
52	copaene	1375	0.05	0.05	0.15	-	0.14	-
53	carvacryl acetate	1391	-	-	-	-	-	0.80
54	methyl eugenol	1398	-	-	-	-	0.14	-
55	calarene	1409	-	0.19	-	-	-	-
56	*α*-humulene	1413	-	0.17	-	-	0.41	-
57	transcaryophyllene	1418	-	-	0.11	2.00	2.30	2.27
58	(+)-aromadendrene	1440	0.21	2.53	-	-	0.02	-
59	*β*-patchoulene	1441	-	-	-	-	0.04	-
60	*β*-elemene	1445	0.17	-	-	-	-	-
61	caryophyllene	1454	-	0.16	-	0.21	-	0.09
62	aromadendrene	1467	1.86	-	-	-	-	-
63	*α*-gurjunene	1475	0.06	0.31	-	-	-	-
64	trans-*β*-farnesene	1479	0.69	-	-	-	-	-
65	*γ*-gurjunene	1481	-	0.31	-	-	-	-
66	*α*-curcumen	1482	-	-	-	0.15	-	-
67	germacrene-D	1485	0.07	-	0.11	-	-	-
68	ledene	1489	-	0.95	-	0.15	-	-
69	*α*-*guaiene*	1491	-	0.04	-	-	-	-
70	*β*-selinene	1493	0.24	0.04	-	-	0.02	-
71	eremophilene	1502	-	0.41	-	-	-	-
72	*α*-muurolene	1504	-	-	-	-	0.03	-
73	*δ*-guaiene	1505	-	1.37	-	-	-	-
74	bicyclogermacrene	1505	1.28	-	-	-	-	-
75	*β*-bisabolene	1506	-	-	0.03	0.41	0.03	-
76	*α*-amorphene	1511	0.05	0.04	-	0.06	0.19	-
77	*γ*-cadinene	1513	-	0.16	-	-	-	-
78	Δ-cadinene	1524	0.20	0.09	0.53	-	0.16	-
79	*β*-sesquiphellandrene	1525	-	-	-	1.34	-	-
80	zingiberene	1526	-	-	-	-	-	-
81	cadina-1,4-diene	1532	-	-	0.13	-	-	-
82	ledol	1560	0.66	-	-	-	-	-
83	(-)-allospathulenol	1576	0.89	-	-	-	-	-
84	spathulenol	1581	13.45	-	-	-	-	-
85	caryophyllene oxide	1582	-	-	0.48	0.54	0.49	-
86	viridiflorol	1587	-	8.14	1.89	-	-	-
87	caryophyllenol-I	1641	-	-	0.34	-	0.36	-
88	t-muurolol	1641	1.03	-	-	-	-	-
89	torreyol	1643	-	-	0.22	-	-	-
90	isospathulenol	1644	1.57	-	-	-	-	-
91	cadalin	1652	-	-	0.15	-	-	-
92	(Z,Z)-farnesal	1720	0.72	-	-	-	-	-
93	trans-farnesol	1722	5.37	-	-	-	-	-
94	farnesal	1738	1.08	-	-	-	-	-
95	farnesyl acetate 3	1825	0.43	-	-	-	-	-

__^a^  Identification of components based on GC-MS Wiley 7.0 version library and National Institute and Technology 05 MS (NIST) library data. _ _^b^  KI: Kovats Indices on HP-5MS capillary column. _ _^c^  River red gum. _ _^d^  Blue gum. _ _^e^  Lavender. _ _^f^  Oregano. _ _^g^  Rosemary. _ _^h^  Thyme.

**Table 2:** Oviposition rates (%OR), hatching of eggs (%E), percentage of reproductive efficiency (%RE), and percent reproduction inhibition (%RI) of female *H.scupense* ticks with different concentrations of essential oils tested.

Essential oil (*μ*l/ml)		OR (%)	E (%)	RE (%)	RI (%)
EC					
	0.097	31.37 ± 7.12 g	65.99 ± 0.03 fe	28.41 ± 3.79 hg	54.71 ± 3.84 g
	0.195	38.54 ± 0.56 hg	62.86 ± 5.74 e	24.24 ± 6.28 gfe	61,37 ± 7.09 hg
	0.390	42.84 ± 3.89 i	61.86 ± 1.18 e	22.19 ± 2.18 f	64.64 ± 5.26 ih
	0.781	52.02 ± 4.42 j	59.42 ± 3.87 dc	17.89 ± 2.19 e	71.49 ± 4.52 jih
	1.562	64.56 ± 9.11 k	54.69 ± 7.88 c	12.16 ± 1.82 d	80.62 ± 1.86 k
	3.125	81.22 ± 0.59 l	8.52 ± 2.69 b	1.00 ± 0.10 b	98.40 ± 0.36 m
	6.25	94.49 ± 1.19 m	0.00 a	0.00 a	100.00 n
	12.50	100.00 n	0.00 a	0.00 a	100.00 n

EG					
	0.097	2.69 ± 0.47 b	99.53 ± 0.14 j	64.71 ± 6.17 nml	3.15 ± 1.48 a
	0.195	4.85 ± 0.28 c	99.38 ± 0.04 j	63.19 ± 6.92 ml	5.43 ± 2.63 ba
	0.390	5.93 ± 1.58 c	98.64 ± 0.17 ih	62.00 ± 4.69 l	7.21 ± 1.92 cb
	0.781	19.20 ± 3.61 d	98.57 ± 0.18 ih	53.22 ± 2.55 kj	20.35 ± 6.77 d
	1.562	21.76 ± 5.91 ed	97.84 ± 1.88 h	51.15 ± 6.09 ji	23.45 ± 8.02 ed
	3.125	36.86 ± 0.53 hg	8.04 ± 2.69 b	3.39 ± 1.18 c	94.92 ± 2.09 l
	6.25	43.02 ± 3.06 i	0.00 a	0.00 a	100.00 n
	12.50	100.00 n	0.00 a	0.00 a	100.00 n

LS					
	0.097	1.53 ± 0.09 a	99.44 ± 0.09 e	65.43 ± 0.08 l	2.08 ± 0.12 a
	0.195	3.09 ± 1.59 a	99.11 ± 0.54 e	64.18 ± 0.49 k	3.95 ± 0.74 b
	0.390	3.23 ± 0.91 a	95.51 ± 1.48 d	61.76 ± 1.35 j	7.57 ± 2.02 c
	0.781	33.36 ± 6.21 ecd	94.21 ± 2.83 d	41.95 ± 1.78 f	37.21 ± 2.66 gi
	1.562	60.01 ± 6.53 g	93.40 ± 3.32 d	24.96 ± 1.25 d	62.65 ± 1.87 igfh
	3.125	99.88 ± 0.01 i	0.00 ± 0.00 a	0.00 ± 0.00 a	100 ± 0.00 l
	6.250	100 ± 0.00 i	0.00 ± 0.00 a	0.00 ± 0.00 a	100 ± 0.00 l
	12.50	100 ± 0.00 i	0.00 ± 0.00 a	0.00 ± 0.00 a	100 ± 0.00 l

OF					
	0.097	10.90 ± 2.46 bc	91.80 ± 2.48 d	54.66 ± 2.09 i	18.21 ± 3.13 d
	0.195	21.15 ± 9.18 cd	90.58 ± 4.58 d	47.73 ± 3.41 g	28.58 ± 5.11 fi
	0.390	41.53 ± 0.57 f	89.68 ± 1.13 d	35.04 ± 0.62 e	47.57 ± 0.93 hi
	0.781	45.48 ± 3.66 f	70.37 ± 7.33 c	25.64 ± 3.78 d	61.64 ± 5.65 i
	1.562	47.98 ± 4.57 f	4.31 ± 0.99 b	1.50 ± 0.49 b	97.76 ± 0.73 k
	3.125	100 ± 0.00 i	0.98 ± 0.01 a	0.00 ± 0.00 a	100 ± 0.00 l
	6.250	100 ± 0.00 i	0.00 ± 0.00 a	0.00 ± 0.00 a	100 ± 0.00 l
	12.50	100 ± 0.00 i	0.00 ± 0.00 a	0.00 ± 0.00 a	100 ± 0.00 l

RO					
	0.097	0.36 ± 0.08 a	97.33 ± 1.18 h	64.80 ± 6.45 nml	3.02 ± 0.17 a
	0.195	21.25 ± 2.64 ed	96.90 ± 0.59 h	50.99 ± 2.09 ji	23.70 ± 2.94 ed
	0.390	23.75 ± 2.00 fe	93.13 ± 2.91 g	47.45 ± 3.56 i	28.99 ± 1.06 fe
	0.781	31.79 ± 3.48 g	95.55 ± 1.01 g	43.55 ± 1.63 i	34,82 ± 1.59 g
	1.562	95.72 ± 1.91 m	0.00 a	0.00 a	100.00 n
	3.125	100.00 n	0.00 a	0.00 a	100.00 n
	6.25	100.00 n	0.00 a	0.00 a	100.00 n
	12.50	100.00 n	0.00 a	0.00 a	100.00 n

TC					
	0.097	9.04 ± 2.33 b	99.77 ± 0.02 e	60.65 ± 0.02 j	9.24 ± 0.03 c
	0.195	21.42 ± 1.09 c	99.57 ± 0.11 e	52.28 ± 0.08 hi	21.76 ± 0.12 e
	0.390	27.45 ± 5.07 de	98.47 ± 0.87 e	47.74 ± 0.60 g	28.55 ± 0.89 fi
	0.781	67.79 ± 0.82 h	98.23 ± 0.66 e	21.14 ± 0.20 c	68.36 ± 0.30 j
	1.562	100 ± 0.00 i	0.00 ± 0.00 a	0.00 ± 0.00 a	100 ± 0.00 l
	3.125	100 ± 0.00 i	0.00 ± 0.00 a	0.00 ± 0.00 a	100 ± 0.00 l
	6.25	100 ± 0.00 i	0.00 ± 0.00 a	0.00 ± 0.00 a	100 ± 0.00 l
	12.50	100 ± 0.00 i	0.00 ± 0.00 a	0.00 ± 0.00 a	100 ± 0.00 l
	Control	-	100 ± 0.00 f	66.82 ± 0.00 m	-

Means within a column followed by the same letter are not significantly different by the Kruskal Wallis test (p ≥ 0.05).

**Table 3:** Lethal concentrations of 50%, 90%, and 95% of *H. scupense* larvae with different doses of essential oils tested.

Essential oil	LC_50_ (*μ*l/ml)		LC_90_ (*μ*l/ml)		LC_95_ (*μ*l/ml)	
(*μ*l/ml)	24 h	6j	24 h	6j	24 h	6j
EC	0.207 a	0.003 a	1.653 b	0.066 a	2.978 b	0.151 a
EG	0.155 a	0.081 c	2.387 b	0.705 a	5.183 c	1.301 b
LS	0.253c	0.039b	2.212c	0.324c	4.092c	0.587c
OF	0.131b	0.030b	0.982b	0.176b	1.740 b	0.290b
RO	0.108 a	0.017 b	0.495 a	0.073 a	0.761 a	0.110 a
TC	0.058a	0.016a	0.358a	0.058a	0.600a	0.083a

Means within a column followed by the same letter are not significantly different by the Tukey test (p ≥ 0.05).

## Data Availability

No data were used to support this study.
